# A cyanidin-3-O-rutinoside dominant anthocyanin system from *Osmanthus fragrans* fruit peel: Composition, color behavior and thermal stability

**DOI:** 10.1016/j.fochx.2026.104392

**Published:** 2026-08-31

**Authors:** Weiyi Xu, Yutong Meng, Fan Yu, Huanfeng Ye, Xuejiao Xu, Jie Chen, Xianrui Liang, Sheng Fang

**Affiliations:** a School of Food Science and Biotechnology*,* Zhejiang Gongshang University, Xuezheng Street No. 18*,* Hangzhou 310018*,* China; b School of Agriculture, Food, and Ecosystem Sciences, Faculty of Science*,* The University of Melbourne*,* Parkville*,* VIC 3010*,* Australia; c College of Pharmaceutical Sciences*,* Zhejiang University of Technology*,* Hangzhou 310014*,* China; d College of Biology and Environmental Engineering*,* Zhejiang Shuren University*,* Hangzhou 310018*,* China

**Keywords:** *Osmanthus fragrans* fruit peel, Cyanidin-3-O-rutinoside, Natural colorant, Molar absorptivity, Thermal degradation kinetics

## Abstract

This study evaluated *Osmanthus fragrans* fruit (OFF) peel as a potential source of anthocyanin-based natural colorants. Anthocyanins were extracted using acidified ethanol and purified by macroporous resin adsorption, yielding a pigment-rich extract with a recovery of 10.4 ± 0.2% from dried peel. HPLC–MS/MS analysis indicated that cyanidin-3-O-rutinoside (C3R) was the predominant anthocyanin in the extract. The molar absorptivity of C3R was experimentally determined to enable more accurate quantification of C3R-rich systems. The purified extract exhibited typical pH-dependent color behavior, maintaining a stable red coloration under acidic conditions (pH 2.0–4.0). Thermal stability experiments showed that C3R degradation was strongly influenced by temperature and pH, and the degradation behavior followed first-order kinetics. In addition, the extract displayed measurable antioxidant capacity in DPPH and FRAP assays. These results demonstrate that *O. fragrans* fruit peel is a promising source of C3R-rich anthocyanins with potential applications as natural colorants in acidic food systems.

## Highlights

• Identification of cyanidin-3-O-rutinoside (C3R) as the dominant anthocyanin.
• Experimental determination of molar absorptivity of C3R for quantification.
• Thermal degradation of C3R characterized by first-order kinetics.
• C3R-rich extract showed stable red coloration under acidic conditions.
• Extract exhibited measurable antioxidant capacity.

## 1 Introduction

*Osmanthus fragrans* is a popular ornamental plant renowned for its distinctive floral scent. It has been widely used in traditional Chinese cuisine and medicine and has been reported to exhibit various bioactive properties, including antioxidant, anti-inflammatory, and anti-platelet aggregation activities (Jeong et al., 2020; Tang et al., 2015; Wang et al., 2010). While its flowers are extensively utilized in foods and beverages, the fruit remains largely underutilized (Han et al., 2021; Yang et al., 2023). During the fruiting season, substantial quantities of mature purple-black fruits fall naturally. This results in the loss of potentially valuable phytochemicals and poses an environmental disposal challenge. A previous transcriptomic study has demonstrated anthocyanin biosynthesis in the pericarp of sweet osmanthus (Han et al., 2021), providing a biochemical basis for exploring the mature purple peel as a potential source of natural pigments.

The increasing consumer demand for natural and clean-label products has intensified the search for plant-derived alternatives to synthetic colorants in the food industry (Morote et al., 2024; Nunes et al., 2024). Anthocyanins, water-soluble flavonoids responsible for the red to purple coloration of many fruits and vegetables, have attracted particular interest as natural colorants. However, a major challenge in the industrial use of anthocyanins lies in the compositional complexity of most natural sources. Many anthocyanin-rich materials contain heterogeneous mixtures of multiple anthocyanins with different stability and color characteristics. For example, blueberries contain numerous anthocyanin derivatives derived from several anthocyanidin aglycones (Zhang et al., 2022), while mulberries typically contain comparable proportions of cyanidin-3-O-glucoside (C3G) and cyanidin-3-O-rutinoside (C3R) (Liang et al., 2023). Such compositional heterogeneity may complicate quality control and hinder the interpretation of pigment stability and functionality. Natural sources with relatively simple anthocyanin compositions may therefore offer advantages for both industrial applications and mechanistic studies. It is known that the spectral properties, molar absorptivity, color response, and thermal stability of anthocyanins are strongly influenced by their molecular structures (Ahmadiani et al., 2016; Sigurdson et al., 2019; Sui et al., 2014). Therefore, a naturally simplified anthocyanin system may allow more reliable quantification and clearer interpretation of structure-dependent color and stability behavior.

Previous studies on pigments from *Osmanthus fragrans* fruits (OFF) have reported inconsistent results. For example, melanin was extracted from the seeds using alkaline methods (Wang et al., 2006), whereas a red compound tentatively identified as salidroside was obtained (Pan et al., 2009). These discrepancies may arise from differences in extraction methods, fruit tissues, and degradation of pigments. In addition to pigment-related compounds, several phenylethanoid glycosides such as salidroside, neonuezhenide, and nuezhenide have also been separated and characterized in the fruits (Hu et al., 2018). Notably, only the peel of mature OFF exhibits a distinct purple-black coloration, suggesting the presence of pigment compounds such as anthocyanins. Meanwhile, previous studies have shown that anthocyanin degradation is highly dependent on molecular structure, pH, and temperature (Hou et al., 2013; Klavins et al., 2026; Sui et al., 2014). However, the mature purple peel has rarely been investigated independently as a natural colorant source, and the identity of its predominant anthocyanin, the appropriate quantitative parameters, and its pH- and temperature-dependent stability remain unclear.

To address this gap, the present study evaluated OFF peel as a potential source of anthocyanin-based natural colorants. The investigation focused on four main aspects: (1) development of an extraction and purification procedure for anthocyanins from the peel; (2) identification of the major anthocyanin components by HPLC–MS/MS and determination of the molar absorptivity of C3R to improve quantification accuracy; (3) characterization of pH-dependent color properties and thermal degradation kinetics of the extract; and (4) evaluation of antioxidant capacity using *in vitro* chemical assays. The results provide insights into the pigment composition, stability, and potential application of OFF peel as a source of natural colorants for acidic food systems. Such naturally simplified anthocyanin systems are rarely reported and may provide useful model systems for investigating anthocyanin stability and color behavior.

## 2 Materials and methods

### 2.1 Materials and chemicals

Mature fruits of *Osmanthus fragrans* ‘Ziyingui’ (Albus Group) were harvested from the Xiasha campus of Zhejiang Gongshang University in Hangzhou, China. The reference standard of Cyanidin-3-O-rutinoside chloride (PCode 102,502,609) was obtained from Sigma-Aldrich (St. Louis, MO, USA). Formic acid, glacial acetic acid, hydrochloric acid, ferric chloride hexahydrate, and methanol were supplied by Shanghai Aladdin Biochemical Technology Co., Ltd. (Shanghai, China). l-Ascorbic acid, gallic acid, ferrous sulfate heptahydrate, 2,2-diphenyl-1-picrylhydrazyl (DPPH), and 1,3,5-tri(2-pyridyl)-2,4,6-triazine were obtained from Macklin Biochemical Co., Ltd. (Shanghai, China). Sodium acetate, ethanol, sodium dihydrogen phosphate dihydrate, and disodium hydrogen phosphate were purchased from Sinopharm Chemical Reagent Co., Ltd. (Shanghai, China).

### 2.2 Extraction and purification

#### 2.2.1 Crude anthocyanin extraction

The peel was manually separated from the fresh fruits carefully, and lyophilized using a Scientz-10 N freeze-dryer (Ningbo Scientz Biotechnology Co., Ltd., China). The dried material was then ground into a fine powder using an A11 Basic analytical mill (IKA, Germany). This procedure yielded 2.9 ± 0.1 g of dried peel powder per 100 g of fresh fruit.

Extraction was performed according to a previously reported method (Han et al., 2024) with minor modifications. Briefly, the peel powder was mixed with 70% (*v*/v) acidified ethanol (pH 2.0, adjusted with HCl) at a solid-to-liquid ratio of 1:20 (*w*/*v*). The mixture was subjected to ultrasonic-assisted extraction (40 kHz, 40 °C) for 30 min, followed by centrifugation at 8000 rpm for 10 min. After extraction, the supernatants were collected, and the extraction solvent was removed by rotary evaporation. The resulting concentrated extract was designated as the crude extract (CE) and subjected to resin purification without drying.

#### 2.2.2 Macroporous resin purification

The concentrated CE (1.1 g, corresponding to approximately 3.0 g of dried peel powder) was further purified following a reported resin-based method for anthocyanins (Zhang et al., 2019) with slight modifications. Briefly, the CE was loaded onto an AB-8 macroporous resin column at a flow rate of 1.5 mL/min. The column was first rinsed with 7 bed volumes (BV) of deionized water to remove impurities such as proteins, sugars, and other polar compounds. Subsequently, the target anthocyanins were eluted using 3 BV of 80% (*v*/v) ethanol. Both steps were performed at a constant flow rate of 1.5 mL/min. The collected ethanolic eluate was concentrated by rotary evaporation at 40 °C and then freeze-dried to obtain the purified extract (PE).

### 2.3 Chemical identification and quantification

#### 2.3.1 HPLC analysis conditions for anthocyanins

An LC-16 HPLC system (Shimadzu, Japan) equipped with a Welch Ultimate LP-C18 column (250 mm × 4.6 mm, 5 μm; Welch Materials, China) was employed for anthocyanin analysis. The column temperature was maintained at 45 °C, and anthocyanins were detected at 520 nm. The mobile phase consisted of (A) methanol and (B) 5% (*v*/v) aqueous formic acid, delivered at a flow rate of 1.0 mL/min. A gradient elution program was applied as follows: 0–2 min, 75% → 70% B; 2–20 min, 70% → 67% B; 20–20.1 min, 67% → 70% B; 20.1–25 min, 70% B. All samples were filtered through a 0.45 μm hydrophilic PTFE membrane prior to injection (10 μL).

#### 2.3.2 HPLC-MS/MS identification

Anthocyanins were identified using a Thermo Scientific UltiMate 3000 HPLC system coupled with a mass spectrometer detector (LCQ Advantage, Thermo, Waltham, MA, USA). Separation was achieved on a Welch Ultimate LP-C18 column (250 mm × 4.6 mm, 5 μm; Welch Materials, China) maintained at 45 °C. The injection volume was 10 μL, and detection was performed at 520 nm. Electrospray ionization (ESI) was operated in positive ion mode with a capillary voltage of 35.49 V. Nitrogen was used as the drying and nebulizing gas at a flow rate of 18.64 L/min and a temperature of 300 °C. The mobile phase consisted of (A) methanol and (B) 0.1% (*v*/v) formic acid in water, with the following gradient program: 0–10 min, 100% → 93% B; 10–20 min, 93% → 88% B; 20–30 min, 88% → 85% B; 30–40 min, 85% B.

#### 2.3.3 Determination of molar absorptivity of C3R

The molar absorptivity (ε) of C3R was determined based on a reported method (Sigurdson et al., 2019) with modifications. A stock solution was prepared by dissolving 1.0 mg of C3R standard in 1 mL of distilled water. This solution was then serially diluted with phosphate buffer (pH 1.0 and pH 4.5) to obtain six different concentration levels. Absorbance was measured at 520 nm and 700 nm using a UV-2600 UV–Vis spectrophotometer (Shimadzu, Japan). The molar absorptivity was calculated according to the Lambert–Beer law, using the following equations:

(1) $$A={\left(A_{520\mathrm{nm}}-A_{700\mathrm{nm}}\right)}_{\mathrm{pH}1.0}-{\left(A_{520\mathrm{nm}}-A_{700\mathrm{nm}}\right)}_{\mathrm{pH}4.5}$$

(2) $$\varepsilon =\frac{A}{c\times L}$$

where *A* is the difference in absorbance between pH 1.0 and 4.5 buffer solutions, ε is the molar absorptivity (L/mol/cm), *c* is the concentration of the C3R solution (mol/L) and *L* is the path length (1 cm).

#### 2.3.4 Quantification of total anthocyanin content (TAC)

The TAC was determined using the pH differential method according to Lu et al. (2020), with slight modifications. Briefly, the extract (1 mg/mL) was diluted 10-fold separately in potassium chloride buffer (pH 1.0) and sodium acetate buffer (pH 4.5). After equilibration for 15 min, the absorbance of each solution was measured at 520 nm and 700 nm using a UV–Vis spectrophotometer. The TAC was calculated as C3R equivalents using the experimentally determined molar absorptivity according to the following equations:

(3) $$\mathrm{TAC}=\frac{A\times \mathrm{MW}\times \mathrm{DF}}{\varepsilon \times L}$$

where *A* is the absorbance difference from eq. (1), *MW* is the molecular weight of C3R (595.98 g/mol), *DF* is the dilution factor, *ε* is the molar absorptivity coefficient of C3R (21,974 L/mol/cm), and *L* is the path length (1 cm).

### 2.4 Colorimetric and stability characterization

#### 2.4.1 pH-dependent color characterization

The pH-dependent colorimetric properties were evaluated by diluting a PE solution (1.0 mg/mL) with phosphate buffer solutions across a pH range of 2.0–12.0. The visual color changes were documented with a digital camera, and the absorption spectra were recorded using a UV-2600 spectrophotometer (Shimadzu, Japan).

#### 2.4.2 Thermal-pH stability studies

The stability of C3R in the PE under different pH and temperature conditions was evaluated according to a previously reported method (Swer & Chauhan, 2019) with slight modifications. PE solutions were mixed with phosphate buffer (50 mL) at pH 2.0, 4.0, and 6.0. Equal aliquots were transferred into screw-capped glass vials and heated in a water bath at 60, 75, and 90 °C. Samples were collected at predetermined time intervals over 6 h, cooled to room temperature, and analyzed by HPLC to determine the remaining C3R content. Colorant retention was expressed as the percentage of the chromatographic peak area relative to the initial value (0 h). All experiments were performed in triplicate.

#### 2.4.3 Kinetic analysis of thermal degradation

The thermal degradation kinetics were modeled using a first-order reaction model (Fang et al., 2019). The degradation rate constant (*k*) and half-life (*t*_1/2_) were calculated using the following equations:

(4) $$\ln\left(C_{0}/C_{t}\right)=k\times t$$

(5) $$t_{1/2}=-\ln\left(0.5\right)/k$$

where *C*_0_ is the initial concentration and *C*_t_ is the concentration at time *t*. The rate constant *k* was derived from the slope of the linear plot of ln(*C*_0_/*C*_t_) *versus* time.

### 2.5 Antioxidant activity evaluation

#### 2.5.1 DPPH radical scavenging assay

The antioxidant activities were evaluated according to Ren et al. (2023), with slight modifications. A 400 μL aliquot of the extract solution (1.0 mg/mL) was mixed with 400 μL of DPPH solution (20 μmol/L in ethanol) and incubated in the dark for 30 min at room temperature. The absorbance was then measured at 517 nm. The radical scavenging activity (%) was calculated accordingly. Ascorbic acid was used as a reference standard.

#### 2.5.2 FRAP assay

The FRAP reagent was freshly prepared by mixing acetate buffer (pH 3.6), 10 mM TPTZ in 40 mM HCl, and 20 mM FeCl₃·6H₂O at a ratio of 10:1:1 (*v*/v/v). A 50 μL aliquot of the purified extract or ascorbic acid standard was mixed with 950 μL of the FRAP reagent and incubated at 37 °C for 30 min. The absorbance was measured at 593 nm using a microplate reader.

### 2.6 Statistical analysis

All experiments were performed three times unless otherwise stated. The values are shown as the mean ± standard deviation. One-way analysis of variance (ANOVA) followed by Tukey's honestly significant difference (HSD) post-hoc test was used to compare group means, and differences were considered significant at *p* < 0.05.

## 3 Results and discussion

### 3.1 Valorization of *Osmanthus fragrans* fruit peel and anthocyanin composition

*Osmanthus fragrans* fruits are widely distributed in East Asia and have attracted increasing attention as sources of bioactive compounds. *O. fragrans* typically blooms in September in Hangzhou, China (30°N, 120°E), producing oval fruits with a distinctive purple peel (Fig. S1). Previous phytochemical studies mainly focused on the whole fruit and reported the presence of iridoid glycosides (Tang et al., 2021) and other phenolic constituents (Tang et al., 2023). However, the peel of the fruit exhibits a distinct purple coloration (Fig. S1c), suggesting the presence of anthocyanin pigments. Valorization of fruit peel as a natural colorant source is of particular interest from both sustainability and food processing perspectives.

#### 3.1.1 Extraction and purification of anthocyanins from OFF peel

**Fig. 1:**
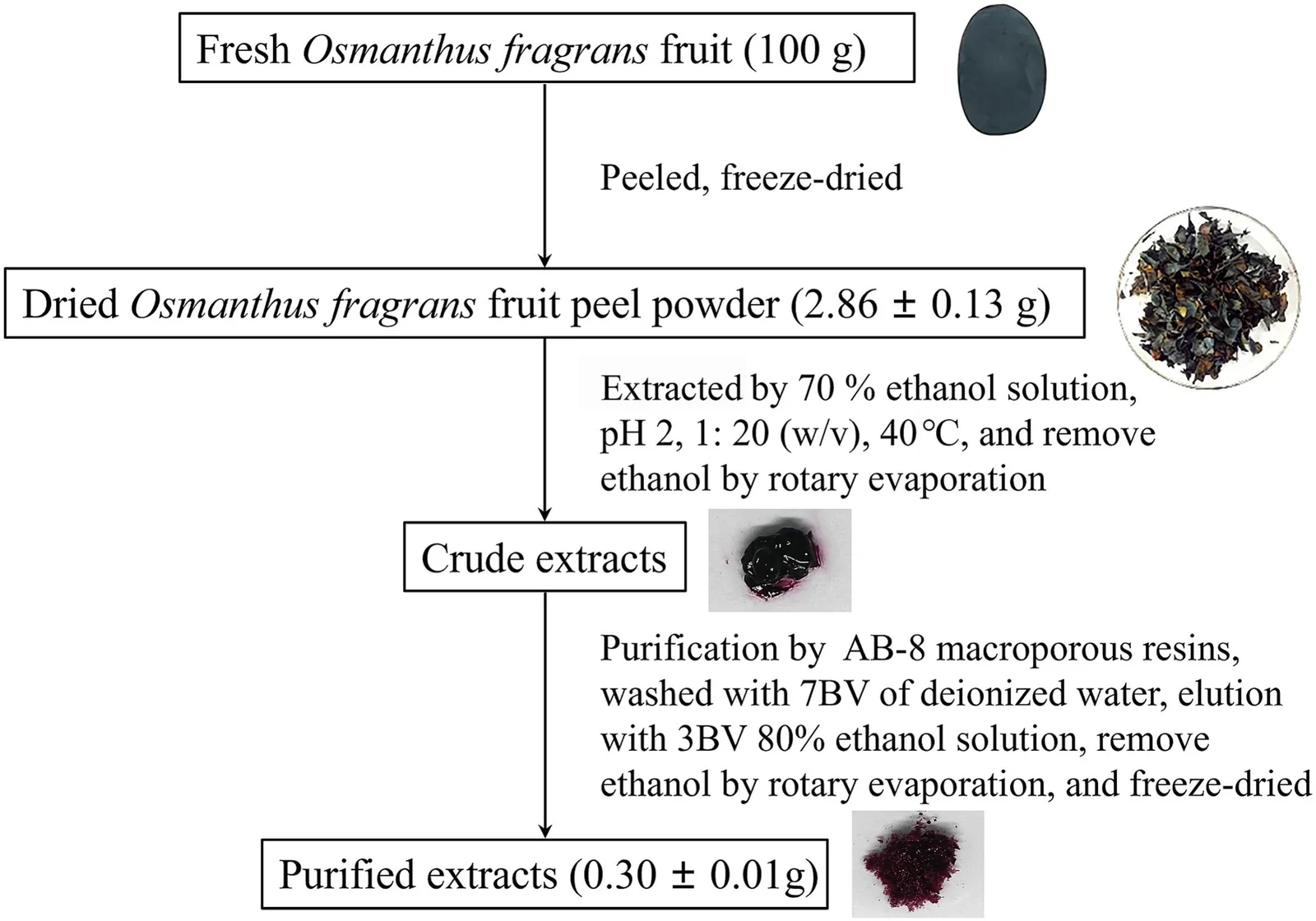
The procedure and amounts for extraction and purification of colorants from *Osmanthus fragrans* fruit peel.

Fig. 1 shows the procedure for extracting and purifying the colorants from OFF peel. Based on our previous studies on anthocyanin extraction from plant materials (Lu et al., 2020; Zhang et al., 2019), acidified aqueous ethanol (70%, *v*/v) was adopted as a practical extraction system for OFF peel. Systematic optimization of the extraction conditions was beyond the scope of the present study. The crude extracts were susceptible to moisture when exposed to air at a relative humidity of 55% and a temperature of 20 °C (Fig. S2a). This may be due to the presence of hydrophilic substances, such as polysaccharides. Therefore, the crude extract was further purified using AB-8 macroporous resin column chromatography to remove polysaccharides and other hydrophilic impurities. The purified extracts (PE) showed less apparent moisture absorption than CE under the same conditions (Fig. S2b), facilitating their storage, handling, and subsequent characterization of pigment properties. As shown in Table 1, after the above process, the total yield of colorants from OFF peel was 10.4 ± 0.2% (colorants/dried peel powder, *w*/w).

**Table 1:** Comparison of yield (%, *w*/w), cyanidin-3-O-rutinoside (C3R) content, and hygroscopicity between crude extract (CE) and purified extract (PE) from *Osmanthus fragrans* fruit peel.

Parameter	CE	PE	Fold increase
Yield (%, *w*/w) a	58.6 ± 1.2	10.4 ± 0.2	/
C3R (mg/g)	28.7 ± 1.8	154.9 ± 4.1	5.4
Moisture uptake	High	Minimal	/

a Yield was calculated based on the weight of dried peel powder.

#### 3.1.2 Anthocyanin composition and identification of the dominant pigment

**Fig. 2:**
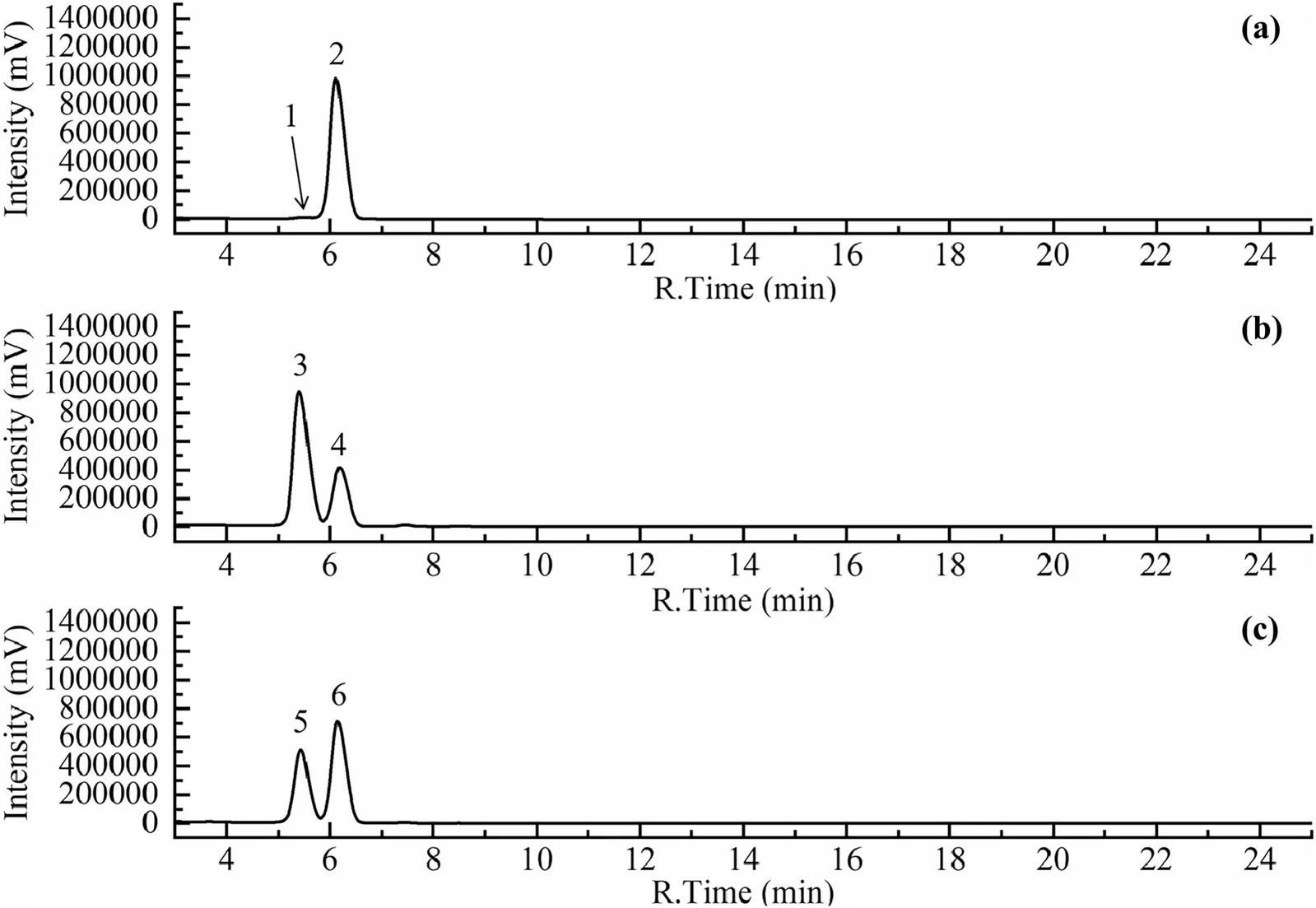
HPLC chromatograms (520 nm) of (a) extracts of *Osmanthus fragrans* fruit peel, (b) extracts of mulberry, and (c) a mixture of these two extracts (*v*/v, 1:1).

HPLC analysis of the purified extract (PE) at 520 nm revealed two peaks (Peak 1 and Peak 2), with Peak 2 representing more than 99% of the detectable anthocyanin signal (Fig. 2a). For preliminary identification, the chromatographic profile of PE was compared with that of well-characterized mulberry anthocyanins. The mulberry extract also exhibited two major peaks (Peak 3 and Peak 4) with retention times nearly identical to those observed for Peaks 1 and 2 in PE (Fig. 2b). Co-injection of a 1:1 (*v*/v) mixture of the two extracts resulted in complete peak overlap (Fig. 2c), suggesting that the two samples may contain similar anthocyanin species. Mulberry anthocyanins are known to comprise cyanidin-3-O-glucoside (C3G) and cyanidin-3-O-rutinoside (C3R), with C3G eluting first (Natić et al., 2015). Accordingly, Peaks 1 and 2 were tentatively identified as C3G and C3R, respectively. It should be noted that the chromatographic analysis targeted typical anthocyanin absorption at 520 nm; therefore, minor degraded derivatives that do not absorb at this wavelength cannot be completely excluded.

The anthocyanin composition was further confirmed by MS/MS analysis. As shown in Fig. S3 and Table 2, Peak 1 (0.8% of total peak area) exhibited characteristic ions at *m*/*z* 449 [M]^+^ and *m*/*z* 287 [M-162]^+^, corresponding to C3G (Fig. S4a) (Yawadio et al., 2007). Peak 2, which represented the predominant anthocyanin signal (99.2%), displayed a molecular ion at *m*/*z* 595 [M]^+^ with fragment ions at *m*/*z* 449 [M-146]^+^ and *m*/*z* 287 [M-146-162]^+^. These sequential losses of 146 and 162 Da correspond to the cleavage of rhamnose and glucose residues from the rutinoside disaccharide, respectively, confirming the structure of C3R (Fig. S4b) (Hou et al., 2013).

**Table 2:** Identification of anthocyanins in *Osmanthus fragrans* fruit peel extracts by HPLC-MS/MS: Peak assignments, retention times (Rt), relative abundances (R) based on peak area, and observed mass-to-charge ratios ([M + H]^+^) of precursor/product ions.

Peak	Compound	Rt (min)	R (%)	[M + H]^+^ (*m*/*z*)
1	Cyanidin-3-O-glucoside	8.02	0.8	449.03, 287.08
2	Cyanidin-3-O-rutinoside	8.67	99.2	595.04, 449.04, 287.12

Compared with many commonly used anthocyanin sources, the anthocyanin composition of OFF peel appears relatively simple. For example, mulberry fruits typically contain both C3G and C3R in comparable proportions (Liang et al., 2023), while blueberries often exhibit more complex anthocyanin profiles comprising multiple glycosylated derivatives (Zhang et al., 2022). The predominance of a single anthocyanin species may offer practical advantages for colorant applications, as a simplified pigment composition can reduce variability in color expression and facilitate the interpretation of stability behavior during processing. In this regard, OFF peel may serve as a useful model system for investigating the color properties and thermal stability of C3R-rich anthocyanin extracts.

#### 3.1.3 Quantification of C3R and determination of molar absorptivity

The pH differential method is widely used for the quantification of total anthocyanins due to its simplicity and robustness. In this method, C3G is commonly used as the reference compound because its molar extinction coefficient (ε = 26,900 L/mol/cm) is well established. However, the accuracy of this approach may be limited when the dominant anthocyanin in the sample differs from C3G (Ahmadiani et al., 2016). For example, C3R represented the predominant anthocyanin detected in the OFF peel extract. Therefore, determining its molar absorptivity is essential for improving the reliability of anthocyanin quantification.

A commercial C3R standard was used to determine its molar extinction coefficient. The spectrum exhibited two characteristic absorptions at 281 nm and 512 nm (Fig. S5a), corresponding to the aromatic ring and the flavylium chromophore, respectively, consistent with previous reports (Vergara et al., 2009). Serial dilutions of the C3R standard (4.41 × 10^−6^ to 4.41 × 10^−5^ mol/L) were prepared in pH 1.0 and 4.5 buffer systems and analyzed using the pH differential procedure. Linear regression based on the Lambert–Beer relationship showed good linearity between absorbance and concentration within the tested range (Fig. S5b).

The molar extinction coefficient of C3R was calculated to be 21,974 L/mol/cm, which is lower than that for C3G. This indicates that using the C3G coefficient to quantify C3R-dominant systems could lead to an underestimation of anthocyanin content by approximately 18%. Based on the new ε value, the anthocyanin content in the OFF peel extract was recalculated. The crude extract contained 28.7 ± 1.8 mg/g dry weight of C3R, while the purified extract reached 154.9 ± 4.1 mg/g, corresponding to approximately 15% (*w*/w) of the dried extract and representing a 5.4-fold enrichment after resin purification (Table 1). It should be noted that the linear relationship obtained here is valid within the tested concentration range. At higher concentrations, deviations from ideal Lambert–Beer behavior may occur due to anthocyanin self-association or solvent effects. Nevertheless, the experimentally determined ε value provides a more appropriate parameter for quantifying C3R-rich extracts and may improve the accuracy of anthocyanin determination in similar matrices (Vergara et al., 2009; Xiao et al., 2024).

### 3.2 Color properties and thermal stability of the C3R-rich extract

#### 3.2.1 pH-dependent color behavior and spectral characteristics

The pH-dependent color behavior of the purified extract (PE) was investigated to evaluate its potential as a natural colorant. As visually shown in Fig. S6a, the solution exhibited distinct color changes across the pH range of 2.0–12.0. The extract appeared bright red under strongly acidic conditions (pH 2.0–3.0), gradually shifted to pink at pH 5.0–6.0, and further changed to greyish-green at pH 7.0–8.0. Under alkaline conditions (pH 9.0–12.0), the color progressively turned brown.

The corresponding UV–Vis spectra at different pH values are shown in Fig. S6b. Under strongly acidic conditions (pH 2.0–4.0), the extract exhibited a maximum absorption wavelength (*λ*_max_) at approximately 514 nm, characteristic of the flavylium cation form of anthocyanins. As the pH increased, the intensity of this absorption gradually decreased and a bathochromic shift of the absorption band was observed, with *λ*_max_ shifting from approximately 524 nm to 570 nm in the pH range of 5.0–9.0. Similar pH-dependent spectral transitions have been reported for anthocyanins from grapes and *Clitoria ternatea* extracts (Nogueira et al., 2024; Yun et al., 2024). These spectral changes reflect the well-known pH-induced structural transformations of anthocyanins, where the flavylium cation predominates under acidic conditions and progressively converts to less stable forms at higher pH, eventually leading to pigment degradation (Levi et al., 2004). Overall, the results indicate that the C3R-rich extract from OFF peel maintains a vivid and stable red coloration under acidic conditions, suggesting that its application as a natural colorant would be most suitable in acidic food systems where both color intensity and pigment stability can be better preserved.

#### 3.2.2 Thermal stability under different pH conditions

**Fig. 3:**
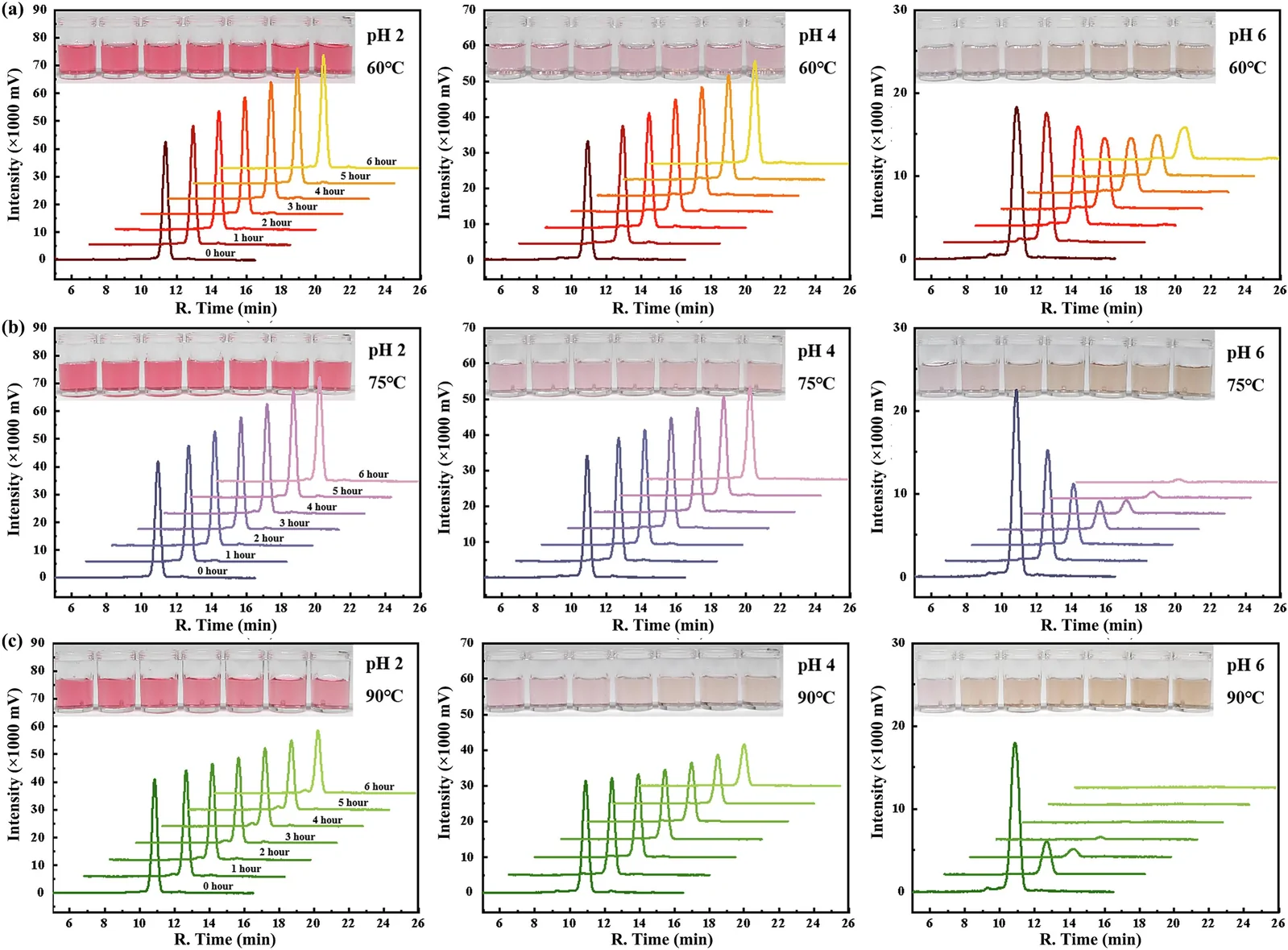
Visual and HPLC chromatographic characterization of cyanidin-3-O-rutinoside (C3R) stability in *Osmanthus fragrans* fruit peel extracts under varying pH and temperature conditions: (a-c) Solution color evolution and corresponding peak profiles at (a) 60 °C, (b) 75 °C, and (c) 90 °C, each demonstrating pH-dependent changes (pH 2.0, left; pH 4.0, middle; pH 6.0, right panels).

The stability of anthocyanins is a critical factor for their application as natural food colorants (Klavins et al., 2026). In this study, the stability of C3R in the purified extract was evaluated under different pH conditions (pH 2.0, 4.0 and 6.0) and temperatures (60, 75 and 90 °C), and the remaining content was monitored by HPLC (Fig. 3). HPLC was used here because it enables specific quantification of intact C3R during thermal degradation, whereas the pH differential method reflects the overall spectrophotometric response of anthocyanins and may also be influenced by degradation products retaining visible absorption. Therefore, HPLC-derived C3R retention values were used for the subsequent kinetic analysis.

Overall, C3R exhibited higher stability under acidic conditions and lower temperatures. At 60 °C and pH 2.0, the degradation of C3R was relatively slow, indicating that the pigment could maintain acceptable stability under mild thermal treatments such as pasteurization. In contrast, increasing temperature and pH markedly accelerated anthocyanin degradation. For example, after 6 h of heating at 90 °C, approximately 55% and 40% of C3R remained at pH 2.0 and pH 4.0, respectively. Under near-neutral conditions (pH 6.0), the degradation was considerably faster. The retention ratios decreased to about 25% at 60 °C and only 2% at 75 °C, while C3R was almost completely degraded after 6 h of heating at 90 °C, as confirmed by the disappearance of the C3R peak in the HPLC chromatogram. The visual color changes observed were consistent with the quantitative HPLC results, further confirming that C3R is the principal pigment responsible for the color of the extract. These observations are consistent with previous reports on anthocyanin stability (da Silva, Reis, Madrona and de Bergamasco, 2025). Non-acylated cyanidin-based anthocyanins are known to be sensitive to elevated temperatures and neutral or alkaline conditions (Klavins et al., 2026). Their degradation may involve cleavage of glycosidic bonds and/or opening of the pyrylium ring with the formation of chalcone-type intermediates, followed by further cleavage into smaller phenolic compounds (Sadilova et al., 2006; Sadilova, Carle and Stintzing, 2007). Protocatechuic acid and phloroglucinaldehyde have been reported as characteristic degradation products derived from the B- and A-rings of cyanidin-based anthocyanins, respectively. Although the degradation products of C3R were not directly identified in the present study, its disappearance under severe heating conditions may involve similar degradation pathways. It should be noted that the stability experiments were conducted using PE rather than CE, because the co-extracted hydrophilic components in CE resulted in pronounced hygroscopicity and complicated its handling and characterization. These components may also influence the color and thermal stability of anthocyanins; therefore, the behavior observed for PE should not be directly extrapolated to CE.

#### 3.2.3 Degradation kinetics of C3R in the extract

**Fig. 4:**
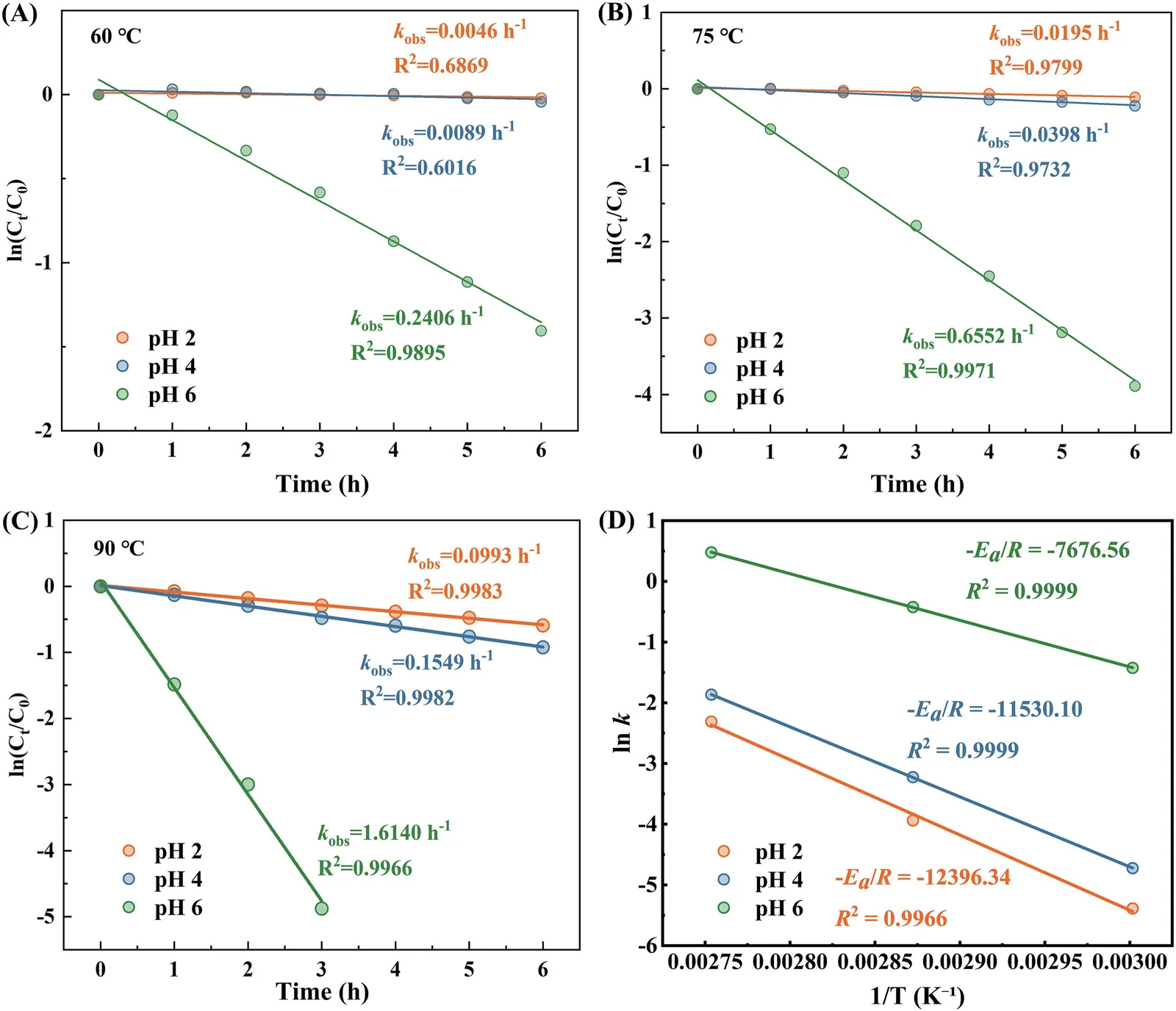
Degradation kinetics of cyanidin-3-O-rutinoside (C3R) in purified extracts (PE) from *Osmanthus fragrans* fruit peel. (a–c) show the degradation profiles at 60, 75 and 90 °C under different pH conditions (2.0, 4.0 and 6.0). The solid lines represent the first-order kinetic models. (d) Arrhenius plots of log(*k*) *versus* 1/*T*, which were used to determine the activation energy (*E*_a_) at each pH.

To quantitatively describe its degradation, the behavior of C3R was modeled using first-order kinetics. The model generally provided satisfactory fits under conditions where appreciable degradation occurred (Fig. 4). However, at 60 °C under pH 2.0 and 4.0, the fits showed relatively low R^2^ values (0.6869 and 0.6016, respectively). This was likely because C3R degradation was limited under these mild acidic conditions during the 6-h experimental period, resulting in concentration changes comparable to experimental variation. Therefore, the kinetic parameters obtained under these two conditions should be regarded as approximate estimates rather than precise kinetic constants. The kinetic parameters are summarized in Table 3. The degradation rate constant (*k*) increased significantly with increasing temperature and pH. For example, under strongly acidic conditions (pH 2.0), the *k* value increased from 0.0046 h^−1^ at 60 °C to 0.0993 h^−1^ at 90 °C. In contrast, degradation proceeded much faster at pH 6.0, where *k* values ranged from 0.2406 h^−1^ to 1.6140 h^−1^ over the same temperature interval. These results indicate that both temperature and pH strongly influence the degradation rate of C3R, with higher temperatures and near-neutral conditions greatly accelerating pigment degradation.

**Table 3:** Degradation kinetics (*k*,*h*^−1^), half-life time (*t*_1/2_, h) and activation energy (*E*_a_, kJ/mol) of Cyanidin-3-O-rutinoside (C3R) in purified extracts (PE) from *Osmanthus fragrans* fruit peel at different temperature and pH.

pH	*k* (h^−1^)			*t*_1/2_ (h)			*E*_a_ (kJ/mol)
	60°C^a^	75 °C	90 °C	60°C	75 °C	90 °C	
2.0	0.0046	0.0195	0.0993	151.67	35.55	6.98	103.06
4.0	0.0089	0.0398	0.1549	78.06	17.42	4.48	95.86
6.0	0.2406	0.6552	1.6140	2.88	1.06	0.43	63.82

a Values at 60°C and pH 2.0–4.0 are approximate estimates because limited degradation during the experimental period resulted in relatively low goodness-of-fit.

The activation energy (*E*_a_) values calculated from the Arrhenius equation decreased from 103 kJ·mol^−1^ at pH 2.0 to 64 kJ·mol^−1^ at pH 6.0, indicating that the degradation rate of C3R became less sensitive to temperature changes as pH increased. However, the substantially higher *k* values at pH 6.0 indicate that C3R degraded much faster overall under near-neutral conditions, despite its lower temperature sensitivity. Comparable trends have been reported for anthocyanins from other plant sources. For instance, Sui et al. (2014) reported *E*_a_ values of 91.5–93.0 kJ·mol^−1^ for C3R degradation in black rice extracts under acidic conditions, decreasing to 66.1 kJ·mol^−1^ at near-neutral pH. The similarity of these values suggests that the thermal degradation behavior of C3R in the OFF peel extract follows patterns consistent with previously reported anthocyanin systems.

To further characterize the thermal resistance of the pigment, the decimal reduction time (D-value) and thermal resistance constant (z-value) were derived from the kinetic parameters (Table S1). The calculated D-values, representing the time required for a 90% reduction in C3R concentration, varied markedly depending on pH and temperature. For example, the D-value was approximately 500 h at pH 2.0 and 60 °C, but decreased to about 1.4 h at pH 6.0 and 90 °C, reflecting the substantial loss of stability under near-neutral conditions. The z-values ranged from approximately 23 °C to 36 °C, indicating that the degradation rate becomes increasingly sensitive to temperature changes at lower pH.

Although the experiments were conducted in buffered model systems, these kinetic parameters provide useful reference information for understanding the thermal behavior of C3R-rich extracts. Overall, the results suggest that acidic environments combined with moderate thermal treatments are more favorable for maintaining the color stability of the extract during food processing.

### 3.3 In vitro antioxidant capacity of the C3R-rich extract

**Fig. 5:**
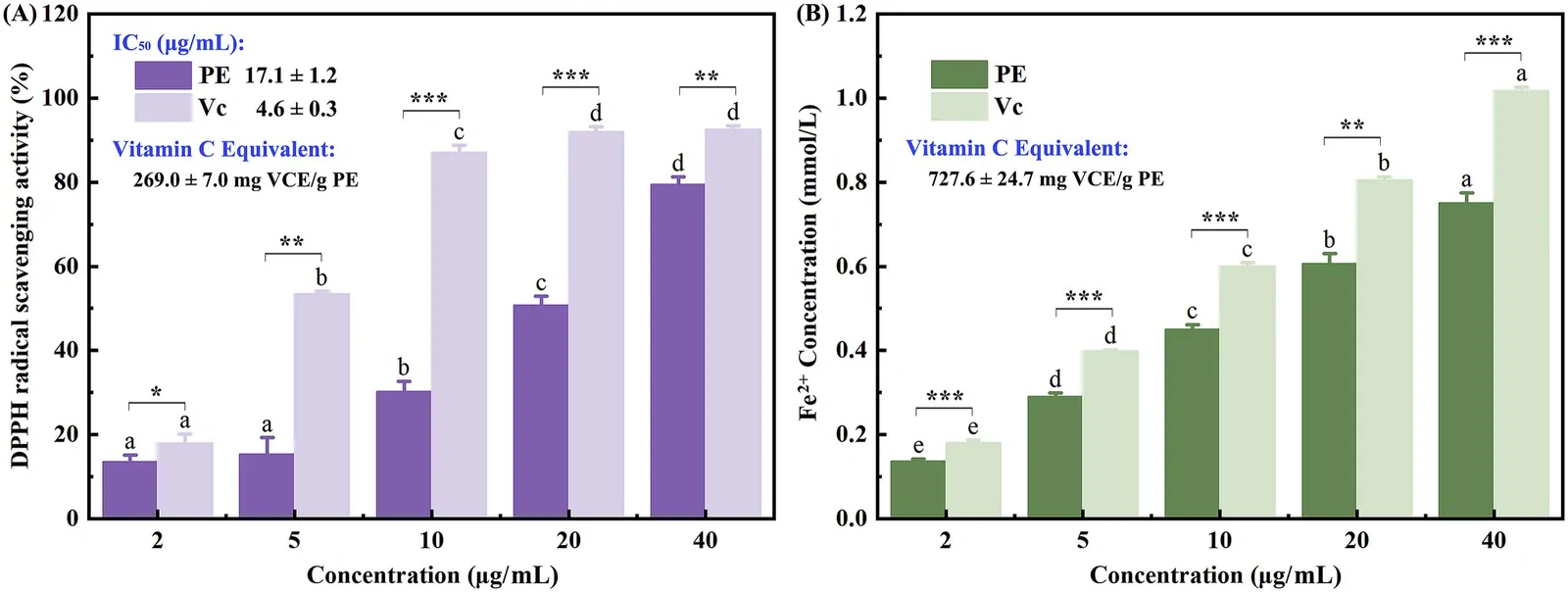
Antioxidant activities of purified extract (PE) from *Osmanthus fragrans* fruit peel. (A) DPPH radical scavenging activity and (B) FRAP reducing power compared with vitamin C (Vc). Different lowercase letters indicate statistically significant differences among concentrations (*p* < 0.05). Asterisks indicate significant differences between PE and Vc at the same concentration (* *p* < 0.05, ** *p* < 0.01, and *** *p* < 0.001).

Fig. 5 shows the DPPH and FRAP activities of the PE and Vitamin C (Vc) used as a reference. The results showed that the extract exhibited notable antioxidant capacity in both assays. When expressed as vitamin C equivalents (VCE), the DPPH scavenging activity of the extract reached 269.0 ± 7.0 mg VCE/g, while the FRAP value was 727.6 ± 24.7 mg VCE/g. The higher value obtained in the FRAP assay suggests that the extract possesses considerable reducing power, which is consistent with the redox properties of anthocyanins. The half-maximal inhibitory concentration (IC₅₀) for DPPH radical scavenging was 17.1 ± 1.2 μg/mL, while the IC₅₀ value derived from the FRAP assay was 62.7 ± 1.5 μg/mL. Anthocyanins are widely recognized for their ability to donate electrons or hydrogen atoms, thereby contributing to radical scavenging and reducing activity.

The antioxidant activity observed for PE is consistent with previous findings for C3R-containing anthocyanin extracts from other plant sources. For comparison, purified C3R from mulberry pomace exhibited a DPPH SC₅₀ of 86.6 ± 2.3 μg/mL (Zhang et al., 2011). C3R, together with cyanidin-3-xylosylrutinoside, was identified as a major contributor to the DPPH, FRAP, and ABTS antioxidant capacities of black raspberries (Tulio et al., 2008). Recently, C3G and C3R were also identified as the major anthocyanins in black mulberry, which exhibited considerable antioxidant capacity in DPPH, ABTS, and FRAP assays (Huo et al., 2023). Although direct quantitative comparison is difficult because of differences in assay conditions and extract composition, these studies collectively demonstrate the substantial antioxidant potential of C3R-containing anthocyanin systems. Beyond direct radical scavenging and reducing capacity, polyphenol-associated antioxidant effects may also involve the regulation of endogenous antioxidant signaling pathways, as reported for other phenolic compounds in studies combining network pharmacology with biological validation (Shen et al., 2026). However, it should be noted that these assays reflect chemical antioxidant capacity under *in vitro* conditions and do not necessarily represent biological antioxidant effects in complex food or physiological systems. Further studies are therefore required to determine whether similar mechanisms contribute to the antioxidant effects of C3R-rich PE.

## 4 Conclusion

This study demonstrates the potential valorization of *O. fragrans* fruit peel as a source of anthocyanin-based natural colorants. An anthocyanin-rich extract was obtained through acidified ethanol extraction and macroporous resin purification, with C3R accounting for 99.2% of the detected anthocyanins. The molar absorptivity of C3R was determined, enabling more accurate quantification of C3R-rich extracts. The color and kinetic analyses further demonstrated that acidic conditions and moderate temperatures are favorable for maintaining the color and stability of C3R. Overall, these results indicate that *O. fragrans* fruit peel is a promising source of C3R-rich natural colorants with potential applications in acidic food systems. Nevertheless, the present stability experiments were mainly conducted in buffered model systems, and the effects of complex food matrices on the color and stability of C3R remain to be established. Future studies should therefore evaluate the performance of the extract in representative food matrices during processing and storage and further characterize the degradation products and pathways of C3R.

## CRediT authorship contribution statement

**Weiyi Xu:** Writing – original draft, Methodology, Investigation. **Yutong Meng:** Writing – original draft, Methodology. **Fan Yu:** Methodology. **Huanfeng Ye:** Validation, Methodology. **Xuejiao Xu:** Validation. **Jie Chen:** Resources. **Xianrui Liang:** Writing – original draft, Supervision. **Sheng Fang:** Writing – review & editing, Supervision, Methodology, Investigation.

## Declaration of competing interest

The authors declare that they have no known competing financial interests or personal relationships that could have appeared to influence the work reported in this paper.

## Data Availability

Data will be made available on request.
