# Supplementary material for: A cyanidin-3-O-rutinoside dominant anthocyanin system from Osmanthus fragrans fruit peel: Composition, color behavior and thermal stability
Source: Food Chem X. 2026 Aug 31;39:104392. doi: 10.1016/j.fochx.2026.104392 (PMC13563582; doi:10.1016/j.fochx.2026.104392)
Supplement: Supplementary file 1 [file mmc1.docx]

**Supporting informations**

**A cyanidin-3-O-rutinoside dominant anthocyanin system from *Osmanthus fragrans* fruit peel: Composition, color behavior and thermal stability**

**Figure S1.** (a) Picture of *Osmanthus fragrans* flowers and fruits during their growing season; (b) Size and removable purple peel; (c) Freeze-dried fruit peel; (d) Purified colorants in solid and water solution.

**Figure S2.** Examples of hygroscopicity of crude extract (a) and purified extract (b) under open air conditions at 20 °C and 55% relative humidity.

**Figure S3.** HPLC-MS/MS of *Osmanthus fragrans* fruit peel extracts: (A) HPLC chromatographic profile and retention times of Peak 1 and Peak 2, (B) MS¹ (top) and MS² (bottom) spectra of Peak 1, and (C) MS¹ and MS² spectra of Peak 2.

**Figure S4.** Structures and MS fragmentation pathways of anthocyanins identified in *Osmanthus fragrans* fruit peel: (a) Cyanidin-3-O-glucoside (C3G, Peak 1) and (b) Cyanidin-3-O-rutinoside (C3R, Peak 2).

**Figure S5**. (a) UV-Visible spectrum of cyanidin-3-O-rutinoside (C3R), exhibiting maximum absorption peaks at 281 nm and 512 nm; (b) Standard curve regressed from concentrations ranging from 4.41×10^-6^ to 4.41×10^-5^ mol/L of C3R, yielding a molar extinction coefficient of ε = 21,974 L/mol/cm based on the Lambert–Beer law.

**Figure S6.** (a) Color variations and (b) UV-Visible spectrum of purified extracts (PE) from Osmanthus fragrans fruit peel at pH levels ranging from 2 to 12.

**Table S1.** Decimal reduction time (D-value) and thermal resistance constant (z-value) for the thermal degradation of cyanidin-3-O-rutinoside (C3R) in purified extracts from *Osmanthus fragrans* fruit peel under different pH conditions.

| pH | D-value (hours) | | | z-value |
| --- | --- | --- | --- | --- |
|  | 60 °C | 75 °C | 90 °C | (°C) |
| 2.0 | 500.65 | 118.1 | 23.19 | 22.54 |
| 4.0 | 258.76 | 57.86 | 14.87 | 24.22 |
| 6.0 | 9.57 | 3.52 | 1.43 | 36.41 |
